# Prognostic, diagnostic and therapeutic value of the cancer tubulin code

**DOI:** 10.1007/s10555-026-10347-w

**Published:** 2026-06-08

**Authors:** Carolina Vilela, Daniela Gomes-Andrade, Sónia Silva, Helder Maiato

**Affiliations:** 1 Faculdade de Medicina, Universidade do Porto, Alameda Prof. Hernâni Monteiro, 4200-319 Porto, Portugal; 2 Chromosome Instability & Dynamics Group, i3S-Instituto de Investigação e Inovação em Saúde, Universidade do Porto, Rua Alfredo Allen 208, 4200-135 Porto, Portugal; 3 IBMC-Instituto de Biologia Molecular e Celular, Universidade do Porto, Rua Alfredo Allen 208, 4200-135 Porto, Portugal; 4 ICBAS-Instituto de Ciências Biomédicas Abel Salazar, Universidade do Porto, R. de Jorge de Viterbo Ferreira 228, 4050-313 Porto, Portugal

**Keywords:** Cancer, Tubulin code, Drug resistance, Microtubules, Microtubule-targeting drugs, Tubulin post-translational modifications, Paclitaxel

## Abstract

Microtubules are essential cytoskeleton polymers composed of α/β-tubulin heterodimers that play a central role in the regulation of various cellular processes, including cell division, cell shape, cell polarity, motility and intracellular trafficking. The expression of different tubulin genes results in a variety of isotypes that, combined with post-translational modifications (PTMs), define a “tubulin code” that generates microtubule diversity. Growing evidence has shown promising links between tubulin isotypes and PTMs with several cancer properties, leading to the emergence of the concept of a “cancer tubulin code”. In this review, we focus on dissecting the impact of tubulin acetylation, detyrosination and polyglutamylation on microtubule properties and functions, and how these PTMs, together with specific tubulin isotypes, affect cancer cell division, invasion and metastasis. Because conventional chemotherapy with microtubule-targeting drugs often leads to resistance and accounts for a high mortality rate among cancer patients, we discuss possible directions that explore the potential of the cancer tubulin code and respective microtubule diversity in improving drug response, while overcoming resistance. Lastly, we address the therapeutic value of small-molecule inhibitors of tubulin-modifying enzymes in cancer treatment. Overall, this review showcases the potential of exploring the cancer tubulin code to open new avenues in diagnostic, prognostic and therapeutic applications for precision oncology.

## Overview of the tubulin code

Microtubules are the largest cytoskeleton components, typically consisting of 13 linear protofilaments laterally arranged in a hollow cylindric structure that has an outer diameter of approximately 25 nm [1–3]. Each protofilament is composed of longitudinally associated heterodimers of globular α-tubulin and β-tubulin molecules [2] that undergo “dynamic instability”, a process by which protofilaments continuously alternate between phases of growth and shrinkage, characterized by transitions between polymerization (growth), catastrophe (depolymerization), and rescue (return to growth) [1, 4, 5]. Furthermore, microtubules exhibit polarity with two distinct ends: a more dynamic plus end capped with GTP bound to β-tubulin, and a less dynamic minus end, often stabilized or capped (*e.g*., by the γ-tubulin ring complex) [1, 6, 7].

Microtubule dynamic behavior, stability and functions are modulated by interactions with canonical microtubule-associated proteins (MAPs), such as Tau, MAP1 and MAP2 that predominantly associate along the length of microtubules; plus-end-tracking proteins (+ TIPs) that transiently bind to the plus end of growing microtubules; and molecular motors that transport cargoes along microtubules at the expense of ATP hydrolysis [8]. Among the latter, dyneins and kinesins carry macromolecules along microtubules, towards the minus end and mostly the plus end, respectively. Moreover, motor proteins generate fundamental forces along microtubules that account for intracellular transport, ciliary beating and self-organization of microtubule arrays (*e.g*. the mitotic spindle) [1, 2, 4].

**Fig. 1:**
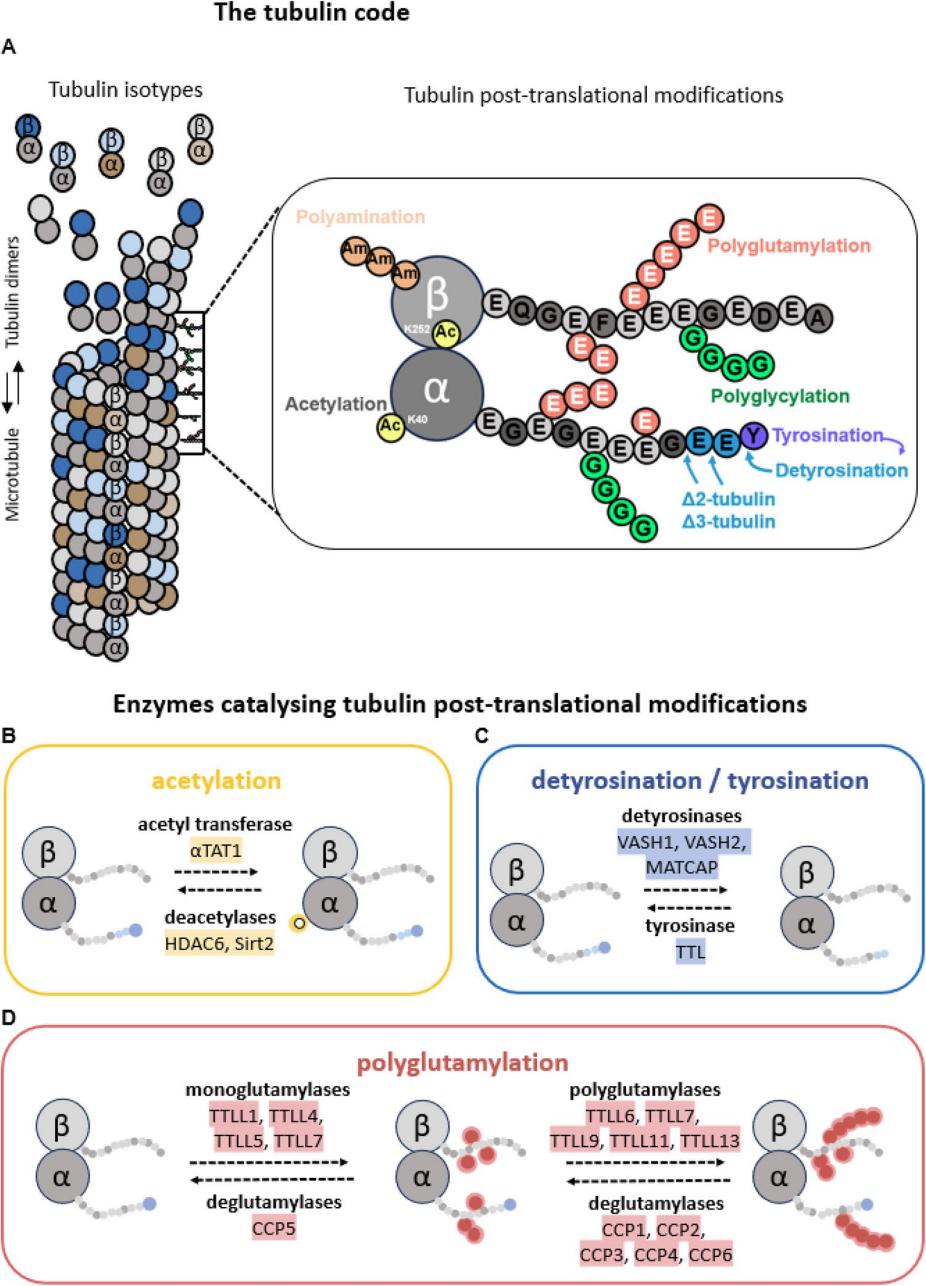
The tubulin code. (**A**) Microtubule diversity is generated through the combination of different α/β-tubulin isotypes and post-translational modifications (PTMs). Tubulin PTMs occur either on defined residues within the tubulin body (*e.g*., acetylation, phosphorylation, polyamination) or along the C-terminal tails (*e.g*., detyrosination/retyrosination, Δ2/Δ3-tubulin formation, polyglutamylation, polyglycylation). (**B**) Enzymes involved in tubulin acetylation and deacetylation. Acetylation of α-tubulin at Lys40, located within the microtubule lumen, is catalyzed by αTAT1, whereas HDAC6 and SIRT2 mediate deacetylation. (**C**) Tubulin detyrosination and tyrosination. Detyrosination of α-tubulin is mediated by VASH1/2 in complex with SVBP and by MATCAP through removal of the C-terminal tyrosine, whereas TTL catalyzes its re-addition. (**D**) Enzymes involved in tubulin polyglutamylation and deglutamylation. Polyglutamylation is catalyzed by TTLL enzymes, which segregate into monoglutamylases (TTLL4, TTLL5 and TTLL7) that initiate side-chain formation, and polyglutamylases (TTLL1, TTLL6, TTLL11 and TTLL13) that elongate glutamate side chains. Deglutamylation is mediated by CCPs. See main text for details. Abbreviations: αTAT1, α-tubulin acetyltransferase 1; CCPs, cytosolic carboxypeptidases; HDAC6, histone/tubulin deacetylase 6; MATCAP, metalloproteinase microtubule-associated tyrosine carboxypeptidase; SIRT2, sirtuin2; TTL, tubulin-tyrosine ligase; TTLL, tubulin-tyrosine ligase-like; VASH1/2, vasohibin 1/2; K40, lysine 40; K252, lysine 252

Most relevant in the context of the present review, microtubules are composed of multiple cell- and tissue-specific α/β-tubulin isotypes that are further combined with post-translational modifications (PTMs) [8, 9]. The resulting tubulin diversity, collectively known as the “tubulin code” (Fig. 1A), can functionally modulate microtubules, regulating their intrinsic properties, as well as interactions with MAPs, + TIPs and motors [6, 9, 10].

Tubulin PTMs such as acetylation, detyrosination, polyglutamylation and polyglycylation are emerging as “fine regulators” of microtubule functions, outlining their potential to significantly disrupt homeostasis and, consequently, have been implicated in several human disorders, including cancer [2, 11]. Among the best studied tubulin PTMs [4], acetylation and detyrosination are specific for α-tubulin, whereas polyglutamylation and polyglycylation may take place on both α- and β-tubulin. While tubulin polyglycylation is essentially confined to stable microtubules in cilia and flagella [12], tubulin acetylation, detyrosination and polyglutamylation are more ubiquitous and can be found on other stable cytoskeletal microtubule populations in different cell types [13–22]. Importantly, none of these tubulin PTMs appear to contribute directly to this enhanced microtubule stability and rather impact a growing list of “readers” that regulate microtubule properties [15]. Here we review how tubulin acetylation, detyrosination and polyglutamylation, together with different tubulin isotypes, affect microtubule functions and shed light into their contribution to cancer-associated properties, with a special focus on drug resistance. Additionally, we evaluate the potential of specific tubulin PTMs as prognostic cancer biomarkers. Lastly, we explore the possibility of selectively targeting tubulin acetylation, detyrosination and polyglutamylation through inhibition of their respective catalytic enzymes as a therapeutic strategy in human cancers, either as an alternative or as a complement to current microtubule-targeting approaches.

### Tubulin isotypes

Cellular microtubule diversity is first expanded by the existence of multiple α- and β-tubulin isotypes, encoded by distinct genes and differing slightly in their amino acid sequence. To date, approximately 8–9 α- and 8–9 β-tubulin isotypes have been identified in humans [23]. Although highly conserved, these isotypes differ primarily within their C-terminal tails, which are also major sites for post-translational modifications, suggesting a functional interplay between isotype composition and PTM patterning [2, 24]. In humans, tubulin isotypes exhibit distinct expression patterns, with certain isoforms, such as αI [25] and αIVa [26, 27], being broadly expressed across tissues, while others show a more restricted distribution, being predominantly found in specialized cell types. These expression patterns are linked to the functional specialization of microtubules in different cellular contexts. Importantly, despite their diversity, tubulin isotypes can incorporate into the same microtubule polymers, forming mixed assemblies that contribute to the fine-tuning of microtubule properties [28].

Functionally, isotype composition influences microtubule dynamics, stability and interactions with microtubule-associated proteins and motor proteins, thereby modulating intracellular transport and cytoskeletal organization [2, 24]. For instance, microtubules enriched in βIII-tubulin display higher dynamicity compared to those composed of βII or βIV isotypes, indicating that isotype composition directly impacts microtubule behavior [29]. Consistently, microtubules assembled from tubulin purified from non-neuronal HEK cells, which predominantly express βI and βIVb isotypes, exhibit faster growth rates and lower catastrophe frequencies than those assembled from brain-derived tubulin [30]. Furthermore, the incorporation of neuronal tubulin into HEK-derived microtubules alters their dynamic properties, suggesting that cells are able to modulate microtubule dynamics by adjusting the relative levels of specific tubulin isotypes [30]. These effects likely result from two main factors: subtle differences in the structured core of tubulin isotypes that affect assembly kinetics and stability, and sequence variations in the C-terminal tails that regulate interactions with different MAPs. Likewise, mutations in tubulin genes can alter tubulin structure and impact microtubule function [6]. Importantly, these structural features also underpin the coordinated interplay between tubulin isotypes and PTMs, as the sequence context of different isotypes can modulate the accessibility and recognition of modification sites, while PTMs further regulate interactions with binding partners.

### Tubulin acetylation

Tubulin acetylation is a highly conserved modification that involves the addition of an acetyl group to Lys40 in α-tubulin, which is located within a loop inside the microtubule lumen (Fig. 1B) [31–33]. Lys40 is acetylated by the highly conserved α-tubulin acetyltransferase αTAT1 [34, 35], which can access the microtubule lumen either through its ends [36, 37] or via defects in the protofilament lattice [36, 38]. Deacetylation of α-tubulin can be performed by histone deacetylase 6 (HDAC6) [39] or sirtuin 2 (SIRT2) [40]. While αTAT1 has only one known cellular substrate (tubulin), HDAC6 and SIRT2 have other substrates, including histones [9]. HDAC6 preferably deacetylates soluble tubulin dimers [41, 42], whereas SIRT2 can impact both soluble tubulin dimers and microtubules [40]. Of note, Lys40 of α-tubulin can also undergo trimethylation by the methyltransferase SET domain-containing 2 (SETD2) [43] and demethylation by KDM4A [44], suggesting that Lys40 of α-tubulin is a critical residue in the regulation of microtubule properties.

Although α-tubulin acetylation is frequently linked to stable/long-lived microtubules [4, 45], its best characterized role is on the mechanical properties of microtubules. Some studies have shown that acetylation at Lys40 changes the structure of a loop in α-tubulin, weakening lateral interactions of Lys60 with His283 from α-tubulins of neighboring protofilaments [2]. These weakened inter-protofilament contacts diminish the flexural rigidity of microtubules, thereby enhancing their flexibility and facilitating protofilament sliding [2, 46]. As such, Lys40 acetylation makes microtubules more resistant to “mechanical aging”, the process by which microtubules lose their flexural rigidity following repetitive bending, preventing their mechanical deformation and prolonging their lifespan in the cell [45, 46]. Interestingly, over 90% reduction of acetylated tubulin after siRNA-mediated depletion of αTAT1 did not significantly affect microtubule half-life in human mitotic spindles [47], and no major differences were observed in microtubule growth rate and length in interphase cells, except for a cell line-specific effect on polymerization duration [47]. Together, these findings indicate that, while α-tubulin Lys40 acetylation can protect microtubules from mechanical stress, this function is highly context- and cell line-dependent. Clearly, more studies are needed to clarify how tubulin acetylation differentially regulates distinct microtubule populations and to define the cellular contexts in which this modification accounts for any measurable change in microtubule dynamics.

### Tubulin detyrosination

Tubulin detyrosination is characterized by the reversible removal of the C-terminal tyrosine from α-tubulin by tubulin carboxypeptidases. Since a C-terminal tyrosine is encoded by the mRNA sequences of most α-tubulin isotypes (TUBA1A/B, TUBA1C, TUBA3C/D/E) [48, 49], the primary post-translational modification is thought to be the catalytic removal of tyrosine, as part of a detyrosination/tyrosination cycle (Fig. 1C) [50, 51]. The first known tubulin carboxypeptidases that detyrosinate microtubules were the cysteine proteases vasohibins VASH1 and VASH2, which depend on the cofactor small vasohibin-binding protein (SVBP) [52, 53]. More recently, other studies identified a third detyrosinase, the metalloproteinase microtubule-associated tyrosine carboxypeptidase (MATCAP) [51], also known as tubulin metallocarboxypeptidase 1 (TMCP1) [54]. Genetic disruption of VASH1, VASH2 and MATCAP resulted in undetectable levels of tubulin detyrosination in cultured cells, suggesting that VASH1/2 and MATCAP enzymes are the major, if not the only, α-tubulin detyrosinating enzymes [51]. Tyrosination, on the other hand, refers to the addition of a C-terminal tyrosine to the α-tubulin detyrosinated tail, and is promoted by a tubulin tyrosine ligase (TTL) [55–58]. Retyrosination depends on TTL concentration, which varies between different tissues and developmental stages [59]. TTL appears to be a highly specific enzyme for tubulin, but it might also be implicated in the regulation of the + TIP protein EB1 (MAPRE1), which has been shown to undergo tyrosination and detyrosination. However, whether this modification on EB1 is directly catalyzed by TTL remains unclear [60, 61]. While TTL preferentially modifies the tubulin heterodimer [58, 62, 63], VASH1/2 acts mostly on the microtubule polymer [52, 64]. Of note, detyrosinated α-tubulin can additionally lose the penultimate and antepenultimate glutamates present in their C-terminal tails and be further converted into Δ2- and Δ3-tubulin, respectively, by the action of cytosolic carboxypeptidases (CCPs) [65, 66]. MATCAP also acts as an α-tubulin detyrosinase, catalyzing the formation of ∆2-tubulin [54]. Because Δ2- and Δ3-tubulin lack at least one critical amino acid in the region that interacts with TTL, these tubulin subtypes are thought not to be further tyrosinated [65], although recent *in vitro* studies have challenged this idea [67].

The interaction between TTL and α/β-tubulin depends on a certain conformation of the dimer, which is disrupted once α/β-tubulin dimers are incorporated into microtubules, thus explaining why TTL is not capable of tyrosinating microtubule polymers [68]. Due to the preference of the detyrosinating carboxypeptidases for microtubules, tubulin detyrosination is associated with longer-lived stable microtubules, whereas more dynamic microtubules are found to be mainly tyrosinated [10, 68, 69]. However, detyrosinated α-tubulin is not responsible for generating microtubule stability *per se* [4, 70], thus being considered a consequence, not a cause, of microtubule stabilization [71]. In fact, experiments using recombinant, engineered human tubulins demonstrated that tyrosinated and detyrosinated microtubules share the same intrinsic dynamics and that the differences in their cellular stability stem solely from differential recruitment of effector proteins [72].

The detyrosination/retyrosination cycle of α-tubulin takes part in important biological processes, including neuronal trafficking, cardiomyocyte beating, cell polarization and cell division [10]. This is achieved by regulating the binding and/or function of several MAPs, + TIPs and motor proteins involved in these processes [2, 73]. Mitotic centromere-associated kinesin (MCAK), and the cytoskeletal-associated protein (CAP)-Gly proteins CLIP170 and dynactin bind preferentially to tyrosinated microtubules [2, 68, 74, 75], while the kinesin motors centromere-associated protein E (CENP-E) and kinesin-2 tend to associate with detyrosinated microtubules [2, 74, 76]. By reducing the binding of CLIP170 and dynactin proteins to microtubules, detyrosination negatively impacts microtubule growth speed and persistence [2]. Additionally, detyrosination has been implicated in the regulation of microtubule flexibility and dynamics through studies on the α4A-tubulin isotype, a genetically encoded detyrosinated α-tubulin isotype. The incorporation of α4A-tubulin into the microtubule lattice stabilizes the polymers against spontaneous and MCAK-driven depolymerization. Because MCAK preferentially depolymerizes tyrosinated microtubules [74, 75], α4A-tubulin-containing microtubules recruit MCAK less efficiently, thereby reducing their depolymerization [77]. These findings underscore the influence of α-tubulin detyrosination in reducing the active disassembly of microtubules by depolymerizing motors of the kinesin-13 family. Given that microtubule stability is tightly linked to the recruitment of effector proteins, understanding how these proteins modulate microtubule-dependent biological processes remains an important area for future research.

### Tubulin polyglutamylation

Tubulin polyglutamylation refers to the attachment of polyglutamate chains to specific glutamate residues within the C-terminal tails of α- and β-tubulin (Fig. 1D) [78–80]. Among the identified modification sites are Glu445 on α1-tubulin (encoded by TUBA1) [78], Glu435 on β2-tubulin (encoded by TUBB2) [80] and Glu438 on β3-tubulin (encoded by TUBB3) [79]. Tubulin polyglutamylation is catalyzed by tubulin tyrosine ligase-like (TTLL) enzymes, a large family of proteins that share a conserved TTL homology domain corresponding to their catalytic core [81, 82]. Each TTLL displays distinct reaction preferences, determined by its accessory domains, including selectivity for α- or β-tubulin, that account for the generation of short or long glutamate side chains [82, 83]. In addition, enzymes catalyzing polyglutamylation preferentially modify microtubules over soluble tubulin dimers [84]. TTLL1, −5, −6, −9, −11 and −13 preferentially glutamylate α-tubulin [82, 85, 86], whereas TTLL4 and −7 glutamylate β-tubulin [82, 83, 85, 87, 88]. Also, TTLL glutamylases segregate into initiating and elongating enzymes: initiating TTLLs catalyze the addition of the initial branch-point glutamate, whereas elongating TTLLs extend the glutamate side chains [9, 85]. In mammals, TTLL1, −4 and −5 act as initiating enzymes, while TTLL6, −9, −11 and −13 function as elongating enzymes. TTLL7 displays both initiating and elongating activities [81, 82, 85, 87–89].

Polyglutamylation is reversed by members of the cytosolic carboxypeptidase (CCP) family (Fig. 1D) [90–92]. These enzymes catalyze the removal of glutamate residues from both the main chain and the side chains of tubulin C-terminal tails, thereby shortening or removing polyglutamate chains, but also generating ∆2-tubulin. Three enzymes (CCP1, CCP4, and CCP6) mediate the shortening of polyglutamate chains, whereas CCP5 specifically removes the branching-point glutamate. In addition, CCP1, CCP4 and CCP6 remove gene-encoded glutamates from the C-terminus [90]. CCP enzymes also exhibit substrate specificity, with distinct preferences for α- or β-tubulin. Together, the coordinated activities of TTLL and CCP enzymes generate complex and dynamic patterns of tubulin polyglutamylation along microtubules [2, 4].

Increasing evidence supports a functional crosstalk between tubulin polyglutamylation and detyrosination, which often co-localize along microtubules. *In vitro* reconstitution experiments demonstrated that overexpression of TTLL5 and TTLL6 are associated with increased levels of detyrosination, since polyglutamylation may promote vasohibin/SVBP-mediated detyrosination in a manner dependent on glutamate chain length [93]. This effect may arise from enhanced interactions between vasohibin/SVBP and the tubulin C-terminal tail, or from increased multivalency and charge density at the microtubule surface, which could facilitate vasohibin diffusion [93]. Reciprocally, TTLL6 exhibits enhanced activity on detyrosinated microtubules [85]. Importantly, *in vivo* studies further demonstrate that detyrosination and polyglutamylation are functionally interdependent, establishing a positive feedback loop in which detyrosination promotes polyglutamylation which, in turn, reinforces detyrosination [94]. Consistently, high levels of tyrosination negatively correlate with polyglutamylation, particularly affecting the elongation of glutamate side chains, whereas reduced tyrosination favors their accumulation [94]. In agreement, loss of vasohibin activity leads to a reduction in polyglutamylation, especially of long glutamate chains, indicating that efficient detyrosination is required for proper polyglutamylation [94]. This highlights that tubulin PTMs are tightly coordinated, with functional crosstalk between distinct modifications contributing to their dynamic regulation.

Tubulin polyglutamylation modulates microtubule dynamics in a chain-length-dependent manner, influencing both microtubule stability and turnover. In cells, polyglutamylation is abundant in long-lived stable microtubules, such as in, but not restricted to, axonemal microtubules and microtubule bundles [95]. Recent *in vitro* reconstitution studies indicate that glutamylation does not directly stabilize microtubules but instead slows the rate of microtubule growth and increases catastrophes [96]. These findings suggest that the relationship between polyglutamylation and microtubule stability *in vivo* is indirect and likely mediated by the recruitment of effector proteins. In this context, polyglutamylation may regulate microtubule dynamics by promoting enzymatic severing mediated by spastin and katanin [22, 97–99]. In HeLa cells, expression of TTLL6, which generates long glutamate side chains, reduced the total microtubule mass, whereas accumulation of TTLL4-catalyzed short side chains had no detectable effect [98]. Similar results were observed *in vitro*, supporting the idea that long glutamate side chains more effectively promote microtubule severing [98].

Despite the enhanced severing associated with long glutamate side chains, highly polyglutamylated microtubules are not constitutively destabilized *in vivo*, suggesting the existence of protective mechanisms. For example, highly polyglutamylated microtubules in axons may be protected by microtubule-associated proteins such as tau, a MAP known to protect microtubules from severing [100]. Polyglutamylation may regulate the binding of tau and other MAPs to microtubules [101, 102], thereby contributing to microtubule stabilization [2, 103]. In fact, the affinity of tau for tubulin is strongly influenced by the extent of polyglutamylation. This modulation is likely driven by conformational changes in the tubulin C-terminal domain induced by increasing chain length [102], linking polyglutamylation to the regulation of intrinsic mechanical properties of microtubules. Accordingly, polyglutamylation increases the stiffness of Taxol-stabilized microtubules by altering interactions between the α-tubulin C-terminal tail and the tubulin body [104]. In addition to regulating interactions with MAPs, polyglutamylation also modulates the activity of molecular motors in a chain length-dependent manner. Experimental systems using tubulin with defined glutamate chain lengths have shown that kinesin motors display differential sensitivity to polyglutamylation patterns. In particular, kinesin-2 motility is induced by chains of three or ten glutamate residues, whereas kinesin-1 requires chains of ten glutamate residues for activation [74]. In contrast, dynein motility and the depolymerizing activity of kinesin-13 appear largely unaffected by glutamate chain length in these *in vitro* reconstitution systems [74], but the situation *in vivo* might be different [105]. These findings suggest that specific patterns and lengths of glutamate side chains can selectively regulate motor protein function. Further studies are needed to define how distinct patterns and degrees of polyglutamylation shape microtubule behavior across different cellular contexts.

## The prognostic and predictive value of the tubulin code in cancer

A wide spectrum of microtubule abnormalities has been reported in numerous cancers, including altered tubulin isotype expression, disrupted tubulin PTMs, and aberrant regulation of MAPs, + TIPs and motors [106]. These alterations have been correlated with cancer aggressiveness, cell migration, metastatic ability, poor patient outcome and sensitivity to chemotherapy (Table 1) [107–110]. Thus, a deeper understanding of the role of tubulin isotypes and PTMs in tumorigenesis may support prognostic assessment and highlight isotype/PTM-dependent vulnerabilities that could be therapeutically explored.

**Table 1:** Involvement of α-tubulin acetylation, detyrosination and polyglutamylation, as well as respective tubulin modifying enzymes in cancer

Tubulin PTM	Alteration	Cancer	Outcome	Refs
Acetylation	Upregulated	Breast cancer	Microtentacle generation, tumour cell reattachment, chemotaxis, and metastatic potential	[107]
		Lung cancer	Epithelial-to-mesenchymal transition via the CAMSAP3/Akt machinery	[199]
		Squamous cell carcinoma of the head and neck (oral cavity tumours)	Higher tumour grade, presence of locoregional lymph node metastases	[307]
	Activated/Overexpressed αTAT1	Colon cancer	Proliferation and invasion via regulation of Wnt1	[200]
		Ameloblastoma	Enhanced migration and invasion	[308]
	Knockdown of αTAT1	Cervical and lung cancer	Reduction in α-tubulin acetylation, compromised actin cytoskeletal integrity and impaired cell growth, cell invasion and migration	[132, 194, 200]
		Breast cancer		
		Colon cancer		
	Overexpressed HDAC6	Glioblastoma	Proliferation, migration, and clonogenicity	[133, 309]
		Cholangiocarcinoma		[134]
	Loss of HDAC6 activity	Lung cancer	Enhanced transport of endosomal vesicles containing EGFR, proliferation and tumour progression	[146]
		Breast cancer		
		Glioblastoma		
	Overexpressed SIRT2	Renal Cell Carcinoma	Poor prognosis, tumour growth and invasiveness	[310]
		Endometrial cancer	Cell stemness, activation of the MEK/ERK signalling pathway, reduced chemosensitivity	[311]
Detyrosination	Down-regulated TTL expression	Breast cancer	Tumour aggressiveness, epithelial-to-mesenchymal transition and metastasis	[108, 201]
		Neuroblastoma	Poor prognosis and patient outcome	[109]
		Ovarian cancer	Tumour development, invasiveness, and drug resistance	[148]
		Prostate cancer	Elevated levels of detyrosinated α- tubulin	[140]
	Suppression of TTL activity	Non-epithelial tumours	Enhanced tumour growth	[147]
	Up-regulation of VASH2	Lung cancer cell line	Epithelial-to-Mesenchymal Transition	[205]
		Breast cancer	Upregulation of growth factors, increased proliferation, epithelial-to-mesenchymal transition	[206, 215]
		Gastrointestinal adenomas and adenocarcinomas	Bigger, irregular and more vascularized tumours	[216]
		Ovarian cancer	Tumour growth, peritoneal dissemination, tumour angiogenesis, chemoresistance to paclitaxel	[217, 218, 312]
		Pancreatic cancer	Epithelial-to-mesenchymal transition, gemcitabine resistance, invasion of tumour cells, upregulation the Hedgehog signaling pathway molecules	[209]
		Cervical cancer	Promoted proliferation, migration, invasion and lymphatic vessel formation and epithelial-to-mesenchymal transition	[210]
		Hepatocellular carcinoma tissues and cell lines	Epithelial-to-mesenchymal transition, increased growth, invasion and angiogenesis	[211, 313]
	Knockdown/deletion of VASH2	Pancreatic ductal adenocarcinoma	Decreased cell migration, reduced liver metastasis and peritoneal dissemination, reduced angiogenesis and reduced recruitment of myeloid derived suppressor cells	[220]
		Triple-negative breast cancer	Inhibited proliferation by delaying cell cycle progression and increasing apoptosis	[207]
		Ovarian cancer	Repressed expression of TGF-β type I receptor, essential for epithelial-to-mesenchymal transition	[208]
Polyglutamylation	Upregulated	Prostate cancer	Estramustine resistance	[140, 314]
		Glioblastoma	Co-localization with MT-associated proteins that promote MT growth and intracellular transport (EB-1/CLIP170/kinesin-1)	[141, 142]
		Breast Cancer	Increased tumour cell proliferation and extracellular matrix remodelling	[143]
	Overexpressed TTLL4	Breast cancer	Brain metastasis, reduced recurrence-free survival, altered extracellular vesicles signature	[138]
	Downregulation of TTLL11	Panel of human cancer cell lines	Defects in chromosome segregation: lagging chromosomes, micronuclei formation	[139]
	Overexpressed TTLL12	Prostate cancer	Inhibition of cell growth, increased DNA content and the number of chromosomes, karyotype instability	[136]
		Ovarian cancer	Correlation with lymph node metastasis, advanced FIGO stage, poor pathological differentiation and reduced overall survival	[137]

In cancer, alterations in tubulin isotype expression have been associated with changes in microtubule behavior, tumor aggressiveness, maintenance of cancer stem cell niches and resistance to microtubule-targeting agents [48, 111]. Changes in the expression of tubulin isotypes have been widely reported across both solid and hematological malignancies, with many tumor types exhibiting upregulation of specific isotypes [112–116]. Notably, the extent of this upregulation often correlates with more aggressive disease and poorer clinical outcomes [112, 114, 117, 118]. Among β-tubulin isotypes, βIII-tubulin, which is normally enriched in neuronal tissues [119], is aberrantly expressed in a broad spectrum of cancers, including breast, lung, gastric, prostate, ovarian, uterine and head-and-neck tumors, and has been consistently associated with poor prognosis and increased metastatic potential [120–125]. Altered expression of other isotypes, such as βI, βII, βIV and βV, has also been described in several cancers, including breast, lung and ovarian tumors [126–131].

Several studies also indicate that α-tubulin acetylation may contribute to cancer progression through diverse mechanisms. However, neither a unifying model nor the cell-type specificity of these effects has been clearly established. This lack of consensus is reflected in contrasting experimental observations across different tumor models. In cervical and lung cancer cells, robust αTAT1 knockdown to reduce α-tubulin acetylation compromised actin cytoskeletal integrity and impaired cell growth [132]. However, other studies have shown that attenuation of α-tubulin acetylation by overexpression of HDAC6 deacetylase promotes the proliferation of glioblastoma cells [133] and cholangiocarcinoma cell lines [134], suggesting a cell-type-specific or a signaling role. As such, rather than exerting a uniform effect on cancer cells, α-tubulin acetylation appears to affect multiple cellular pathways.

Furthermore, α-tubulin detyrosination is well established as a regulator of microtubule dynamics, mitotic processes and directed cell migrations [24, 135] (see next section). However, its contribution to cancer progression remains incompletely understood.

Altered levels of tubulin polyglutamylation, together with differential expression of its regulatory enzymes, have been reported in several cancer types and have been associated with tumor cell proliferation, invasion and metastatic potential. In fact, expression of the polyglutamylase TTLL12 increases during prostate cancer progression and is highly expressed in metastatic lesions, local recurrent tumors and prostatic intraepithelial neoplasia, a precursor of prostate cancer [136]. These observations indicate that deregulated TTLL12 expression may provide a selective advantage during tumor progression [136]. Similarly, TTLL12 expression is significantly upregulated in ovarian cancer tissues and cell lines, where it correlates with lymph node metastasis, advanced FIGO stage, poor pathological differentiation and positive peritoneal cytology [137]. High TTLL12 expression is associated with reduced overall and disease-free survival, supporting a link with aggressive tumor features [137]. Moreover, increased TTLL4 expression promotes β-tubulin polyglutamylation, contributing to brain metastasis, reduced recurrence-free survival and increased extracellular vesicles in breast cancer, indicating that microtubule polyglutamylation directly influences tumor behavior and supports metastatic niche formation through alterations in extracellular vesicle composition [138]. TTLL11 is consistently downregulated in human tumors [139]. As TTLL11 is essential for chromosome segregation fidelity, its downregulation leads to aneuploidy in daughter cells, including chromosome segregation defects such as lagging chromosomes and micronuclei [139]. Therefore, TTLL11 may be part of a cancer-associated signature. The systematic downregulation of TTLL11 in human cancers correlates with reduced polyglutamylation of spindle microtubules, which may contribute to chromosome mis-segregation, aneuploidy and chromosomal instability in tumor cells [139]. Additionally, increased levels of polyglutamylated tubulin have been observed in prostate cancer [140] and in glioblastoma [141, 142]. Also, microtubule glutamylation is upregulated in breast cancer and has been associated with increased tumor cell proliferation and extracellular matrix remodeling [143]. These observations indicate that tubulin polyglutamylation can influence tumor behavior, but its effects appear to be highly context-dependent and enzyme-specific. Although direct evidence linking tubulin polyglutamylation to cancer remains limited, emerging data suggest that this modification may contribute to tumorigenesis by regulating mitotic fidelity, centrosome function and microtubule-dependent transport processes. However, the molecular mechanisms underlying these associations remain poorly understood. Therefore, further investigation into the roles of tubulin polyglutamylation, as well as the specific activities of its modifying enzymes, is required to clarify their contribution to tumorigenesis and to determine whether this PTM can be exploited as a diagnostic or prognostic biomarker.

## How does the tubulin code impact cancer-associated processes?

We next discuss the main mechanisms that have been proposed to explain how the tubulin code impacts cancer-associated processes.

### Cell survival and proliferation

Α-tubulin acetylation regulates the binding of Hsp90 isoforms to tubulin [144]. This enhances the signaling pathways of Hsp90 client proteins, including Akt/PKB, which promote cell proliferation and survival via the PI3K-Akt-NF-kB pathway [144]. Because Hsp90 is overexpressed in many cancers, these findings suggest that α-tubulin acetylation, by promoting Hsp90 recruitment to microtubules (and consequent downstream effects), may contribute to cancer progression, highlighting the therapeutic potential of tubulin acetylation and Hsp90 inhibitors in cancer treatment.

Increased α-tubulin acetylation due to loss of HDAC6 activity also promotes microtubule-dependent transport of endosomal vesicles containing the epidermal growth factor receptor (EGFR) [145], a known driver of tumorigenesis, especially in lung and breast cancer, but also in glioblastoma [146]. Accordingly, the role of α-tubulin acetylation in post-endocytic EGFR trafficking may determine the duration of EGFR signaling and thereby influence cancer cell proliferation and tumor progression [145].

Studies in animal models and human cancers showed that TTL activity is commonly lost during tumor growth, suggesting that TTL suppression and the resulting tubulin detyrosination may confer a selective advantage for proliferating tumor cells [8, 147]. Indeed, low levels of TTL, and consequently reduced tyrosinated α-tubulin, are associated with increased tumor development, invasiveness, and drug resistance [148]. Related to this, excessive α-tubulin detyrosination on kinetochore microtubules may disrupt chromosome congression and segregation, promoting merotelic attachments and, subsequently, chromosomal instability, a hallmark of human cancers [149, 150]. For instance, CENP-E-dependent transport/congression of pole-proximal chromosomes to the metaphase plate depends on the spatiotemporal distribution of tyrosinated and detyrosinated microtubules in the spindle [76]. The Kinesin-13 proteins Kif2b and MCAK’s microtubule-depolymerizing activity at centromeres/kinetochores is suppressed by α-tubulin detyrosination on kinetochore microtubules, resulting in an increase in chromosome segregation errors [149]. In this regard, it was recently proposed that α-tubulin tyrosination can influence MCAK activity in two ways: directly by enhancing its microtubule-depolymerizing activity, or indirectly by facilitating its recruitment to polyglutamylated microtubules [105]. Lastly, the detyrosination/tyrosination status of α-tubulin at kinetochore-microtubule plus-ends fine-tunes CLASP2 and NDC80 at the kinetochore-microtubule interface, ensuring proper chromosome oscillations and accurate timing of anaphase onset [151].

In the context of cell division, polyglutamylation is enriched on spindle microtubules and midbody during cytokinesis, suggesting a role in the regulation of spindle dynamics and mitotic progression [98, 152]. Furthermore, centriolar microtubules within centrosomes, microtubule-organizing centers essential for mitotic spindle bipolarity [153], display high levels of polyglutamylation [21, 154], which is required for centrosome integrity and proper centriole function. Notably, polyglutamylation is not uniformly distributed along centrioles but instead shows distinct domain-specific patterns, suggesting that it may contribute to the spatial organization of centriole-associated proteins within these structures [155, 156]. In line with this, loss of TTLL1 and TTLL9 affects the formation of microtubule bundles and interferes with basal body positioning at the plasma membrane, while defects in glutamylation also lead to abnormal axoneme assembly and altered microtubule-based structures [157]. Also, downregulation of polyglutamylation in dividing cells using antiglutamylation antibodies [158] contributed to centriole disassembly, abnormalities in centriole duplication and cell cycle defects [154, 159], processes that are tightly linked to chromosome segregation fidelity and cell cycle progression. A study using mice spermatozoids lacking Agbl5, showed that the absence of the deglutamylase CCP5 results in supernumerary centrioles, suggesting that glutamylation could control centriole duplication [160]. Given that precise control of centriole duplication is essential for maintaining genomic stability, defects in this process can lead to centrosome amplification, aberrant mitotic spindle formation and chromosomal instability, all of which are hallmarks of cancer [161]. TTLL11-silenced HeLa cells undergoing mitosis also showed a significant increase in chromosome segregation defects, including lagging chromosomes [139]. Tubulin polyglutamylation promotes the activity of microtubule-severing enzymes such as spastin and katanin [22, 97–99], which are involved in regulating spindle dynamics, including microtubule poleward flux [162], spindle length [163] and orientation [164, 165]. In addition, polyglutamylated tubulin is highly concentrated at the midbody [98], where the severing enzymes spastin and katanin are required for the abscission step of cytokinesis [166–168]. Together, these observations highlight the central role of polyglutamylation in regulating microtubule-dependent processes, including mitotic fidelity, centrosome function and intracellular transport, thereby contributing to cellular homeostasis.

### Stress adaptation

Α-tubulin acetylation exerts a protective role against ER stress in cancer cells [169]. Indeed, loss of microtubule acetylation due to αTAT1 suppression disrupts ER homeostasis, activating the unfolded protein response (UPR) and, consequently, apoptosis via the IRE1α pathway. In parallel, α-tubulin acetylation has emerged as a possible dynamic modification in response to various environmental stresses, supporting cellular repair and adaptation to adverse conditions [170]. In this regard, α-tubulin acetylation was shown to rapidly increase upon a wide range of environmental and intracellular stresses, such as ultraviolet exposure and nutrient deprivation, DNA damage [171], oxidative stress [172, 173], and osmotic imbalance, while loss of the acetyltransferase αTAT1 was found to compromise cell survival and genetic repair capacity under stress conditions [171, 172]. Consequently, increased α-tubulin acetylation has been associated with enhanced cell survival and stress tolerance, suggesting a protective role through microtubule stabilization and regulation of intracellular trafficking. However, it remains unclear how cellular stress induces α-tubulin acetylation and facilitates adaptation to cellular damage. Currently, there is no direct evidence linking stress-triggered α-tubulin acetylation to tumor-cell survival or oncogenic transformation, nor demonstrating that it confers tolerance to the chronic stress conditions characteristic of the tumor-microenvironment, including dysregulated immune signalling, intense angiogenesis, accelerated proliferation and enhanced EMT [174]. Understanding whether many standard anticancer therapies that induce DNA damage, such as radiotherapy, etoposide, platinum compounds and reactive oxygen species-generating drugs, enhance α-tubulin acetylation may be highly relevant in order to determine how stress-enhanced α-tubulin acetylation influences treatment response.

Emerging evidence indicates that tubulin polyglutamylation can be regulated in cancer cells through specific signaling pathways linking endoplasmic reticulum stress to mitotic control [175]. In this context, the XBP1 variant Xv1 undergoes unconventional splicing mediated by IRE1α and constitutively by IRE1β, generating the active transcription factor Xv1s [175]. Unlike canonical XBP1, Xv1s does not regulate the unfolded protein response but instead directly upregulates the expression of the polyglutamylase TTLL6 by binding to its promoter. Increased TTLL6 expression promotes microtubule polyglutamylation at the mitotic spindle, where it is enriched on interpolar and kinetochore microtubules [175]. Functionally, this pathway contributes to proper spindle organization, chromosome alignment and successful mitotic progression. Consistently, depletion of Xv1 or TTLL6 reduces spindle polyglutamylation, disrupts interpolar microtubules, impairs chromosome congression and leads to mitotic arrest and cell death in cancer cells [175]. These findings identify an IRE1–Xv1–TTLL6 axis that specifically regulates mitotic spindle polyglutamylation and supports proliferation of cancer cells.

### Cytoskeletal remodelling

The Rho GTPase signaling pathway has emerged as an important driver of tumor cell migration, invasion, and metastasis through the cytoskeletal network [176, 177]. Many cancer types have shown elevated expression of Rho GTPase genes, which correlate with an increased invasive and metastatic phenotype [176]. Detyrosinated α-tubulin promotes Rho/GEF-driven actin stress fiber assembly and cell motility *in vitro* [178]. In addition, the microtubule minus-end-binding protein CAMSAP3 stabilizes non-centrosomal microtubules by anchoring to their minus ends, thereby contributing to cytoskeletal organization [178]. CAMSAP3 loss shifts the microtubule population toward centrosomal arrays that are more extensively detyrosinated. This elevated detyrosination liberates GEF-H1, whose activity is inhibited by binding to microtubules. Subsequent activation of RhoA signaling enhances actin stress fiber formation and reinforces actomyosin contractility, promoting cytoskeletal rearrangements associated with increased cell motility and potentially metastatic behavior [178]. These findings highlight the possible interplay between α-tubulin detyrosination and RhoA signaling in regulating cytoskeletal dynamics during cancer progression, migration and metastasis.

### Cell migration, polarity and invasion

Α-tubulin acetylation has been implicated in cell migration and invasion [179, 180]. Studies in astrocytes revealed that microtubules play an important role in cell migration by modulating cell polarity and the turnover of cell adhesive structures within the extracellular matrix [181]. In this regard, αTAT1-dependent microtubule acetylation localizes to focal adhesion sites, and attenuation of αTAT1 activity reduces cell migration speed without altering migration direction or overall movement. Moreover, αTAT1 was shown to regulate focal adhesion turnover by facilitating the fusion of Rab6-positive vesicles at focal adhesion sites [181]. These results support a pro-migratory role for α-tubulin Lys40 acetylation. Lys40 acetylation may also play a role in cell migration by regulating mechanosensation [35, 182], the process by which cells sense and respond to mechanical cues from their environment, adjust cytoskeletal organization, adhesion, and contractility. These may enhance directional motility, which, in the context of cancer, may increase the metastatic potential [183]. Other studies, including in mouse models, have shown that αTAT1 deficiency or pharmacological inhibition of α-tubulin acetylation caused only mild phenotypic alterations that nevertheless included a decreased touch sensation [182, 184–190], in line with a role of α-tubulin acetylation in mechanosensation.

Interestingly, α-tubulin acetylation and CLASP proteins concentrate at regions of microtubule damage in response to tensile and contractile forces encountered during migration [191]. This localized repair and reinforcement stabilizes the cytoskeleton and allows cellular and nuclear deformation necessary for passage through constricted environments [191]. Moreover, local microtubule acetylation promotes GEF-H1 release and activation underlying actin polymerization, to enable cell contractility and migration forces at focal adhesions [192, 193]. Other studies have shown that αTAT1 localizes to invadopodia in breast cancer cells, where it regulates MT1-MMP metalloproteinases-positive vesicle trafficking and promotes invasive migration through collagen matrices [194]. These findings highlight the crucial role of the acetylation/deacetylation cycle in coordinating cytoskeletal dynamics and matrix degradation, reinforcing its potential as driver of cancer cell migration and metastasis [195]. Indeed, high levels of acetylated α-tubulin correlate with increased metastatic potential in breast cancer cells [107].

Microtubule detyrosination also promotes cell polarization, an essential step for directed cell migration. The trigger for cell polarization is the asymmetric distribution of detyrosinated microtubules: more detyrosinated near the centrosomes, where the migrating edge initiates [135]. Indeed, microtubule detyrosination promotes cell polarization by guiding kinesin-1-mediated transport of the APC protein to specific regions of the cell cortex [135]. In colorectal cancer cells, APC promotes actin filament nucleation, essential for directional migration [196]. In addition, APC-mediated actin nucleation, in cooperation with formins and the Arp2/3 complex, contributes to the formation of invasive protrusions in tumor cells, a key step in the initiation of metastasis [197].

### Epithelial-to-mesenchymal transition

Acetylated α-tubulin has also been proposed to regulate the epithelial-to-mesenchymal transition (EMT), during which epithelial cells lose polarity and junctions and acquire mesenchymal morphology and motility [198]. Accordingly, acetylated α-tubulin promotes EMT in lung carcinoma cells via the CAMSAP3/Akt machinery [199]. CAMSAP3 preserves the epithelial phenotypes in lung cancer cells. The loss of CAMSAP3 results in increased levels of α-tubulin acetylation, leading to the upregulation of Akt proteins activity, which thereby promotes EMT [199]. Reduction of α-tubulin acetylation through αTAT1 knockout impaired cell invasion and migration by attenuating Wnt/β-catenin signaling [200] and MMP-9 expression [194], supporting a role for α-tubulin acetylation in metastasis. In agreement, enhanced α-tubulin acetylation in metastatic breast cancer cells promoted the generation of microtentacles and increased their capacity for reattachment, which are key steps in the metastatic cascade [107].

As for α-tubulin acetylation, α-tubulin detyrosination may be considered a marker of EMT. The induction of EMT downregulates TTL and significantly increases α-tubulin detyrosination, altering microtubule stability and organization to promote plasma-membrane microtentacles [201]. Mesenchymal-like morphology enhances cell motility and metastatic potential, while increasing cellular resistance to mechanical stress [202]. After epithelial cell detachment, cytoplasmic microtubules express high levels of detyrosinated tubulin, stabilizing the polymers. By associating preferentially with more resilient cytoskeletal components, such as the vimentin intermediate filament, detyrosinated microtubules allow for longer lifespan [203]. As such, following detachment, vimentin-expressing invasive breast carcinomas exhibit higher levels of detyrosinated α-tubulin-enriched microtentacles than non-vimentin-expressing non-invasive cell lines, facilitating circulating cancer cells’ reattachment [203, 204]. Also, in lung cancer cells it has been shown that inhibition of α-tubulin detyrosination reverts EMT by reducing vimentin and N-cadherin levels and increasing E-cadherin levels [205]. Moreover, vasohibins have been implicated in EMT in various cancers, including breast [206, 207], ovarian [208], pancreatic [209], cervical [210] and hepatocellular [211] by regulating detyrosination levels and E-cadherin expression. Together, these data indicate that dysregulation of the α-tubulin detyrosination/tyrosination cycle contributes to cancer progression and point to detyrosinated α-tubulin as a potential therapeutic target in the metastatic cascade.

### Angiogenesis

The tubulin carboxypeptidases VASH1 and VASH2 and their associated peptide SVBP were originally recognized as vascular endothelial growth factor (VEGF)-induced secretory proteins involved in angiogenesis, a well-known necessary step for metastasis. Although their roles in cancer have been explored, their precise contribution to tubulin detyrosination-dependent cancer phenotypes remains incompletely understood. VASH1 accumulates in endothelial cells within the termination zone, where it inhibits angiogenesis, whereas VASH2 is expressed in infiltrating mononuclear cells at the sprouting front, promoting angiogenic activity [212]. Indeed, in mouse xenograft models, VASH1 exhibited potent antiangiogenic and anti-lymphangiogenic effects, suppressing tumor lymphangiogenesis and lymph node metastasis [213]. Furthermore, in an *in vivo* xenograft model of lung cancer, VASH1 blocked sprouting angiogenesis by tumors, matured the remaining vessels, and enhanced the anti-tumor effect of conventional chemotherapy [214]. In contrast, VASH2 seems to be a mediator of cancer proliferation [215], promoting tumor growth and angiogenesis [216–218]. Additionally, activity-impairing mutations in VASH1 and VASH2 have been identified in various human carcinomas [219, 220]. More recently, VASH1 overexpression was shown to impair VEGF receptor 2 (VEGFR2) endocytosis and trafficking, leading to reduced signal transduction and cell migration, suggesting that α-tubulin detyrosination negatively regulates angiogenesis [221]. In addition, work in murine pancreatic cancer cells has highlighted the role of VASH2 in regulating cancer cell behavior by increasing α-tubulin detyrosination, a process essential for cell migration [220]. Taken together, these findings highlight the opposing roles of VASH1 and VASH2 in cancer, with VASH1 acting as an anti-tumor and anti-angiogenic factor, whereas VASH2 promotes tumor growth and angiogenesis. The distinct regulation of α-tubulin detyrosination by these enzymes may underlie their opposing effects on tumor angiogenesis and progression. However, further studies are needed to clarify whether these effects are directly mediated by changes in α-tubulin detyrosination or involve additional, context-dependent signaling pathways.

## Drug resistance and the therapeutic value of the tubulin code in cancer

**Fig. 2:**
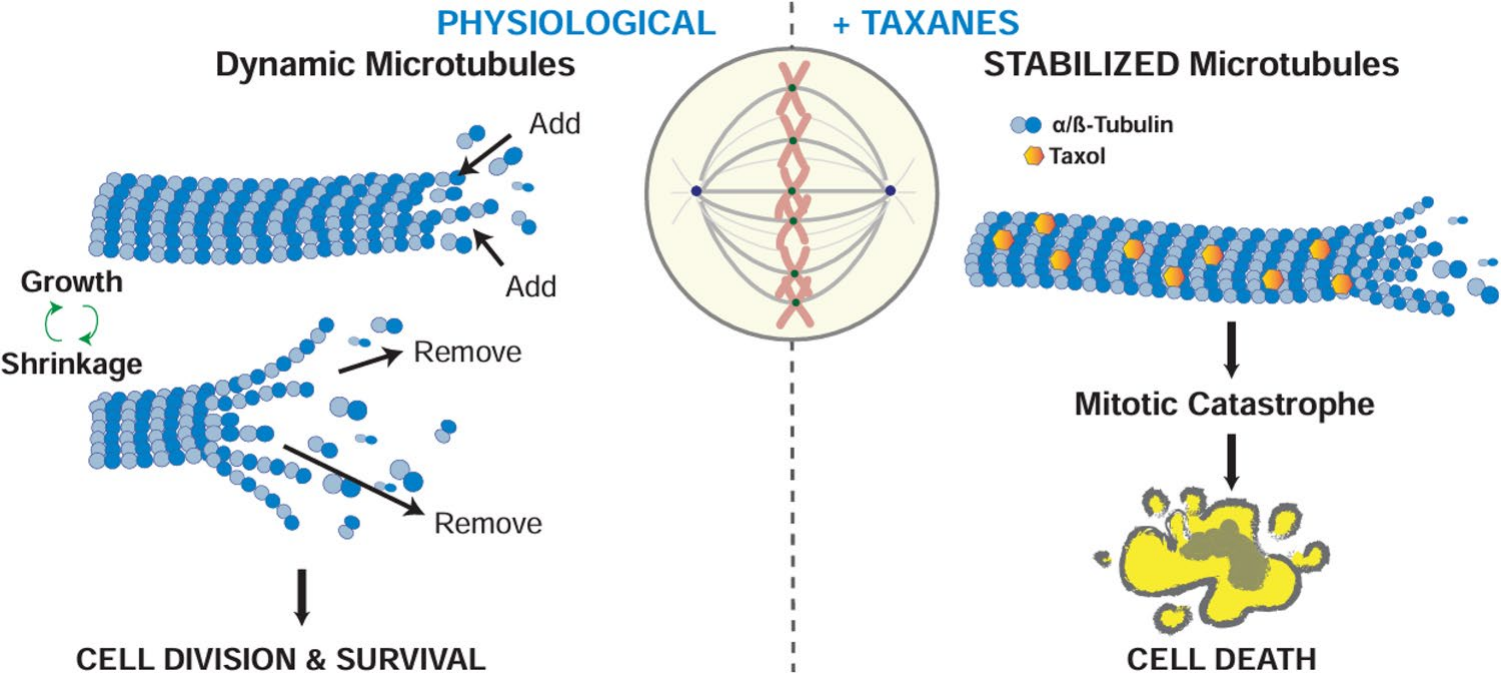
Schematic representation of the mechanism of action of Taxanes. To exert their essential physiological functions, microtubules need to be dynamic structures, undergoing polymerization (growth) and shrinkage (depolymerization) cycles. Taxanes bind to microtubules and suppress their intrinsic dynamic behavior by impeding depolymerization. This results in disruption of microtubule-dependent cellular transport, multipolar spindles, and suppression of spindle dynamics during mitosis, ultimately leading to catastrophic mitotic exit and apoptosis in the subsequent cell cycle

Microtubules have long been used as prime targets of chemotherapeutic agents due to their significant role in cell division and cell migration [7, 222]. As such, several microtubule-targeting agents (MTAs) or tubulin-binding agents (TBAs) are essential in cancer therapy by disrupting cell division and microtubule dynamics [7, 222]. MTAs can be classified in two major groups: microtubule-polymerizing/stabilizing agents, including paclitaxel, docetaxel and epothilones, and microtubule-depolymerizing agents, such as vinka alkaloids, dolastatin, combretastatin and 2-methoxyestradiol [223, 224]. By suppressing microtubule dynamics, MTAs induce chromosomal instability via multipolar mitotic spindle formation without centrosome amplification, delaying satisfaction of the spindle assembly checkpoint, causing a catastrophic exit from mitosis and leading cells to eventually die by apoptosis in the subsequent cell cycle [225–228] (Fig. 2). Because MTAs preferentially affect rapidly proliferating cells, cancer cells become main targets, since they lack the regulatory feedback mechanisms that often restrain cell division, thus maximizing their therapeutic efficacy [229]. However, this remains a matter of debate [225, 230].

Taxanes, including paclitaxel and its semisynthetic analog docetaxel, bind directly to tubulin’s β-subunit on the inside of microtubule surface, inducing a conformational change. This conformational modification stabilizes microtubules and inhibits their disassembly during cell division, thereby promoting cell death [231]. Owing to their effective ability to stimulate microtubule polymerization, taxanes play a key role in the therapy of several tumors, ranging from breast and ovarian cancer, non-small-cell lung cancer to Kaposi’s sarcoma [7]. Although therapeutically useful, its use leads to various severe side effects like neurotoxicity and myelosuppression [7]. As a result, careful use is required to ensure that the therapeutic benefits will unquestionably outweigh the potential harm from side effects.

The emergence of resistance to MTAs represents a major challenge in cancer therapy. Several mechanisms have been proposed to underlie the development of chemoresistance, including the overexpression of ABC transporters and drug efflux pumps [232, 233], tubulin mutations, alterations in tubulin isotype composition or in tubulin-binding proteins [234–236], as well as insufficient activation of cell death pathways [237, 238]. Modifications in MTA binding sites can also alter the activity of these drugs, potentially contributing to chemotherapy resistance [229].

Alterations in tubulin isotype expression have been closely linked to resistance to microtubule-targeting agents. Increased levels of several β-tubulin isotypes, including βI, βII, βIII, βIV and βV, have been correlated with decreased responsiveness to taxanes across different tumor types [121, 129, 239, 240]. Among these, βIII-tubulin has been the most thoroughly investigated, with its overexpression conferring resistance particularly to taxane-based therapies [113, 114, 120–124]. These findings suggest that βIII-tubulin expression levels could be a useful predictive biomarker for response to taxanes. Mechanistically, microtubules enriched in βIII-tubulin display higher dynamicity [29] and reduced sensitivity to paclitaxel compared to those composed of other isotypes [241], suggesting that βIII-tubulin counteracts the microtubule-stabilizing effects of these agents. Consistently, depletion of βIII-tubulin enhances chemosensitivity to a broad range of chemotherapeutic drugs [121, 240, 242], suggesting that βIII overexpression may support cancer cell survival under stress conditions. However, some studies have reported context-dependent effects [243, 244], indicating that βIII-tubulin may differentially influence sensitivity to distinct microtubule-targeting agents. The role of tubulin isotypes in chemoresistance has also driven the identification and use of microtubule-targeting agents that are insensitive to isotype overexpression [245, 246]. Agents such as ixabepilone [247], cabazitaxel [248] and vinblastine [249] have demonstrated increased potency in βIII-tubulin-expressing tumor cells, indicating that targeting isotype-dependent microtubule dynamics may represent a promising strategy to circumvent chemotherapy resistance. These findings highlight the clinical relevance of tubulin isotype composition and support the development of more selective therapeutic approaches.

Tubulin PTMs, namely acetylation, detyrosination and polyglutamylation, have also emerged as key indicators of chemotherapy sensitivity, as they appear to modulate tumor’s response to treatment. Specifically, high α-tubulin acetylation has been suggested as a potential predictive biomarker for paclitaxel therapeutic response in cancer patients [47]. A phosphoproteomic screening of clinical trial samples from HER2-negative, treatment-naive breast cancer patients treated with either paclitaxel monotherapy or a combination of paclitaxel and the multikinase inhibitor nintedanib identified candidate biomarkers of paclitaxel sensitivity. These findings revealed CDK4 and filamin A as accurate predictors of pathologic complete response (pCR) and, consequently, of paclitaxel sensitivity [250]. Patients with high expression levels of both proteins achieved up to 90% pCR following paclitaxel-based chemotherapy [250]. In fact, CDK4 upregulates filamin A, which enhances CLIP170 binding to filamin A-tubulin complexes. This in turn promotes microtubule acetylation and stabilization, thereby increasing paclitaxel affinity for microtubules. Together, these effects lead to mitotic delay and mitotic catastrophe, explaining the enhanced sensitivity to paclitaxel. In contrast, filamin A depletion reduces paclitaxel binding and tubulin acetylation, reverting the increased sensitivity to paclitaxel [250].

A recent study has highlighted the enzymatic mechanism by which α-tubulin acetyltransferases modify Lys40 in the microtubule lumen. Tubulin acetyltransferases interact with the luminal lattice docking its catalytic core onto two α-tubulins and the enzyme’s C-terminal domain overlapping the taxane-binding pockets of two β-tubulins [38]. This spatial and structural interplay suggests a potential mechanism by which microtubule acetylation could regulate taxane cytotoxicity. Another study demonstrated that, within the NCI-60 cancer cell panel, cell lines exhibiting higher levels of α-tubulin acetylation correlated with greater cytotoxicity and enhanced sensitivity to paclitaxel treatment [47]. However, in some cancer cell lines α-tubulin acetylation appears to be dispensable for paclitaxel response. Nevertheless, the use of α-tubulin acetylation as a predictive biomarker may enable the stratification of cancer patients based on their likelihood of responding to paclitaxel, paving the way for more personalized therapies with MTAs, while carefully weighing the potential side effects of the treatment.

Other studies have shown that HDAC6 inhibitors, including the HDAC6-selective inhibitor citarinostat and the broad-spectrum inhibitor valproic acid, enhance the anticancer activity of paclitaxel, producing synergistic effects in various solid tumor lineages, including pancreatic, ovarian and breast cancer cell lines [251, 252]. HDAC6 inhibitors appear to increase α-tubulin acetylation and microtubule stability, thereby reinforcing the tubulin-stabilizing effects of taxane chemotherapy and promoting increased cell death in preclinical models [253], underscoring the benefits of developing future combination therapies for cancer patients. The combination of vorinostat, a potent pan-HDAC inhibitor, with carboplatin and paclitaxel in previously untreated stage IIIb or IV non-small cell lung cancer significantly improved progression-free and overall survival compared with carboplatin plus paclitaxel alone [254]. Likewise, in stage IIa–IIIc breast cancer patients eligible for neoadjuvant chemotherapy, combination therapy with vorinostat and paclitaxel, followed by doxorubicin and cyclophosphamide, and transtuzumab in HER2-positive cases, significantly increased the pathological complete response, supporting a possible role of HDAC activity in enhancing paclitaxel cytotoxicity [255]. These findings highlight the importance of investigating the molecular mechanisms underlying this synergistic effect, particularly the role of HDAC6-mediated α-tubulin acetylation in enhancing paclitaxel cytotoxicity. Indeed, increased α-tubulin acetylation has been associated with greater microtubule stability, which may potentiate the effects of taxanes that target microtubule dynamics. In this context, inhibition of HDAC6 represents a plausible mechanism to reinforce paclitaxel-induced microtubule stabilization and promote cancer cell death. However, given that many of the compounds used in these studies are broad-spectrum HDAC inhibitors, the extent to which these effects can be specifically attributed to HDAC6-mediated tubulin acetylation remains unclear. This distinction is particularly relevant when considering the clinical development of HDAC6-targeted strategies. Further studies using selective HDAC6 inhibitors will be essential to determine whether HDAC6 inhibition is sufficient to enhance paclitaxel sensitivity, and to define its potential as an adjuvant to neoadjuvant chemotherapy.

**Fig. 3:**
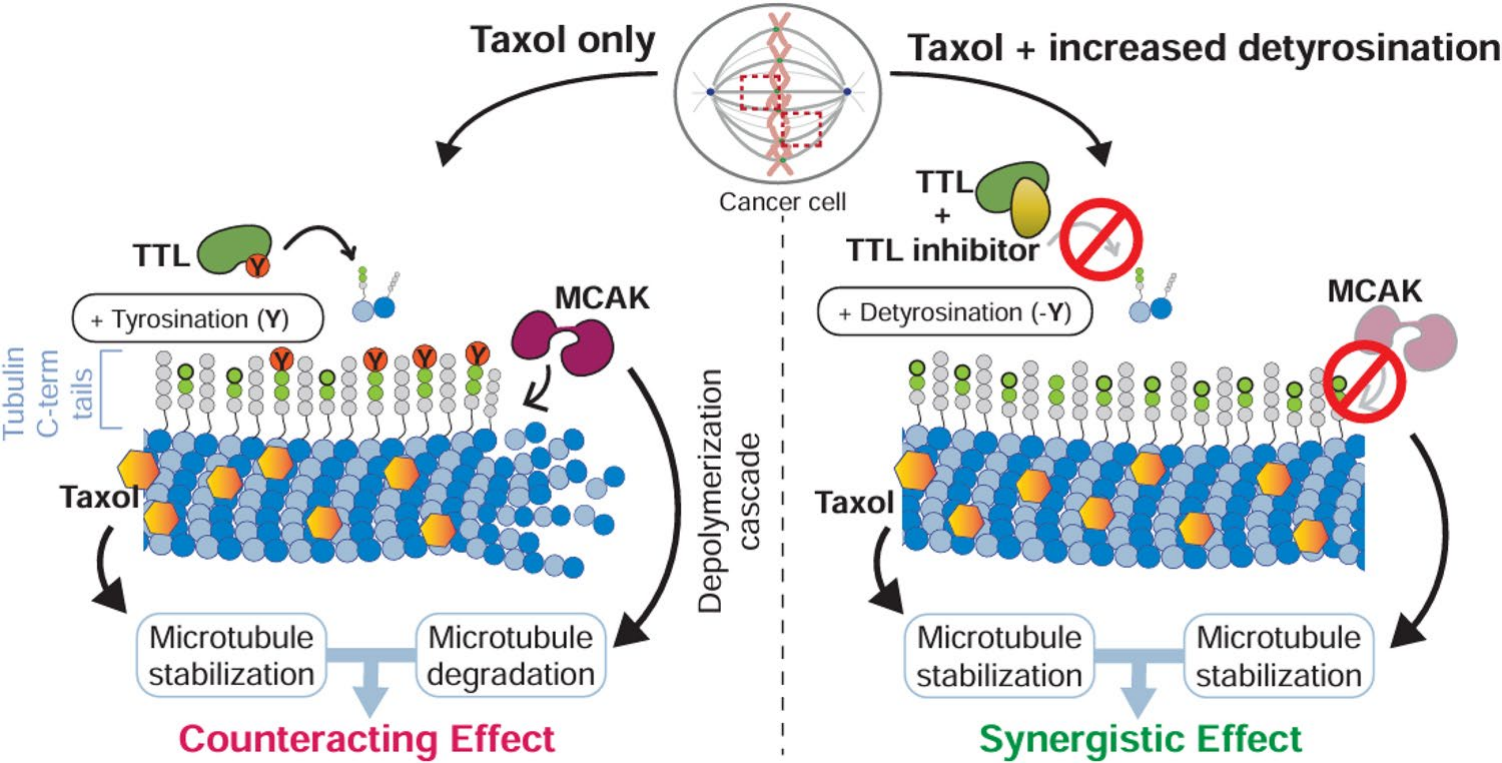
Increased α-tubulin detyrosination, through loss of TTL activity, may promote paclitaxel sensitivity by inhibiting MCAK activity. MCAK can exert its depolymerase function in taxane-stabilized microtubules, counteracting their effect and thus reducing taxane cytotoxicity. Increasing tubulin detyrosination by inhibiting TTL can reduce both the recruitment and depolymerization activity of MCAK on taxane-stabilized microtubules, yielding a synergistic effect when combining TTL inhibition and taxanes

Additionally, α-tubulin detyrosination may be a potential target to modulate the therapeutic window of taxanes, since microtubule detyrosination promotes paclitaxel sensitivity [47]. Indeed, VASH1 overexpression or TTL knockdown in paclitaxel-sensitive cancer cell lines (colorectal, breast and ovarian cancer cell lines) enhanced paclitaxel cytotoxicity by suppressing the activity of the microtubule-depolymerizing enzyme MCAK, while promoting cell death in mitosis and in the subsequent interphase [47, 256]. Indeed, MCAK depletion correlates with increased paclitaxel sensitivity [105], whereas the selective MCAK inhibitor 7S9 prevents MCAK dissociation and release from tubulin and restores microtubule sensitivity to paclitaxel in chemoresistant cells [105]. Observed cellular effects include prolonged mitosis, cytokinesis failure and mitotic cell death, supporting the idea that combining 7S9 with paclitaxel may induce synthetic lethality in chemoresistant cells, enhancing paclitaxel anti-tumor effects [105]. This synergistic interaction is observed not only with paclitaxel, but also with other widely used MTAs, including docetaxel, ixabepilone, eribulin, and vinorelbine, reducing cross-resistance [105]. Thus, by targeting the enzymes that regulate or “read” the detyrosination/tyrosination cycle, rather than microtubules themselves, these selective inhibitors offer a potentially less toxic approach to modulate microtubule activity and may be used with paclitaxel to potentiate its therapeutic effects (Fig. 3).

In addition to acetylation and detyrosination, tubulin polyglutamylation has also been implicated in the modulation of response to microtubule-targeting agents. Increased levels of polyglutamylated tubulin have been identified as a key PTM associated with paclitaxel resistance in breast cancer cells, particularly in triple-negative breast cancer (TNBC) models [105], suggesting that this modification may facilitate the adaptation of cancer cells to MTAs. A recent study demonstrated that MCAK is upregulated in chemoresistant TNBC cells and contributes to paclitaxel resistance and cell migration by preferentially depolymerizing polyglutamylated microtubules [105]. Notably, polyglutamylation enhances MCAK-binding affinity through electrostatic interactions, thereby promoting microtubule depolymerization even in the presence of paclitaxel [105]. Tubulin tyrosination may play a direct role in this process by enhancing MCAK activity, and indirectly, by enhancing polyglutamylation, which promotes MCAK recruitment to MTs, highlighting the functional interplay between these PTMs in cancer resistance [105]. Polyglutamylation may promote chemoresistance by enhancing microtubule dynamic instability through MCAK-mediated depolymerization, thereby counteracting the stabilizing effects of taxanes. These findings suggest that MCAK may serve as a predictive biomarker of breast cancer progression and a potential pan-cancer therapeutic target since it is elevated in multiple solid tumors. 

Septins have also emerged as important regulators of microtubule dynamics and polyglutamylation in the context of taxane resistance. In taxane-resistant breast cancer cells, elevated expression and recruitment of septins, particularly SEPT2, 7, 8, 9 and 11, to microtubules correlate with increased levels of tyrosinated tubulin and long-chain polyglutamylation [257]. Indeed, high levels of tyrosinated tubulin favor the association of septin filaments, especially those containing the SEPT9_i1 isoform, to microtubules bearing short glutamate side chains [257]. Upon binding to the microtubule lattice, septins facilitate the recruitment of polyglutamylation enzymes, including elongating TTLLs and the deglutamylase CCP1, thereby regulating the length of polyglutamate chains [257]. The resulting enrichment of long glutamate side chains, together with septin filaments, promotes the association of microtubule-associated proteins such as CLIP-170 and the depolymerizing kinesin MCAK, which respectively enhance rescue and catastrophe events and ultimately increase microtubule dynamic instability [257]. This shift towards higher microtubule dynamics counteracts the stabilizing effects of taxanes and contributes to drug resistance [257]. Consistently, overexpression of filament-forming septins, particularly those containing SEPT9_i1, was sufficient to induce paclitaxel resistance, an effect that was further enhanced by increased tubulin polyglutamylation [258]. In addition, this resistance was associated with the relocalization of septin filaments from actin stress fibers to microtubules upon paclitaxel exposure [258]. Together, these findings support a model in which septins and polyglutamylation cooperate to modulate microtubule dynamics and promote cellular adaptation to taxane treatment [257, 258].

## Pharmacological manipulation of the tubulin code in cancer

Pharmacological agents targeting “writer” and “eraser” enzymes of the tubulin PTM code, particularly acetylation/deacetylation, tyrosination/detyrosination, and polyglutamylation/deglutamylation, are attracting growing interest in oncology, driven by their roles in microtubule stability and function. Even though detyrosination was first described over 40 years ago [259], the specific enzymes and their structures were only recently elucidated [52, 260–262], lagging behind earlier-characterized acetylation [35, 39], tyrosination [56] and polyglutamylation systems [263]. This temporal gap, convoluted by HDAC6’s superior chemical tractability versus druggability/selectivity hurdles for VASH1/TTLLs/TTL, yields divergent clinical trajectories: deacetylation inhibitors such as ricolinostat have progressed to phase I/II trials, while other tubulin-code enzyme inhibitors remain mainly at discovery and preclinical study phases, currently serving as a primary tool for understanding biological consequences of inhibition.

### Inhibitors of the tubulin acetylation/deacetylation cycle

Numerous HDAC inhibitors have reached the market, but early efforts were focused on pan-HDAC inhibitors which lack isoform selectivity and therefore also modulate histone acetylation and gene expression. Among the best known of this class are hydroxamic acids like suberoylanilide hydroxamic acid (SAHA/vorinostat), trichostatin A (TSA), and the short-chain fatty acid valproic acid (VPA) [264]. These first-generation inhibitor compounds have been evaluated in multiple phase I/II trials, in combination with paclitaxel and other chemotherapies. SAHA is the first FDA-approved HDAC inhibitor for cutaneous T-cell lymphoma [265] and is also used in clinical trials for other cancer types [266, 267]; TSA has entered early-phase tolerability studies in relapsed/refractory hematologic malignancies [268]; and VPA has been assessed in phase I/II trials combined with epirubicin-based chemotherapy in solid tumors [269, 270]. However, the lack of isoform selectivity due to their broad HDAC-inhibition activity leads to adverse side effects in clinical practice such as cardiotoxicity, thrombocytopenia, and bleeding in the gastrointestinal tract [271, 272].

To address the lack of isoform selectivity, in 2003, the first HDAC6-selective inhibitor, tubacin, was discovered via a cell-based high-throughput screening using cytoblot assays with antibodies specific for α-tubulin acetylation and histone acetylation, enabling selective identification from a deacetylase-biased 1,3-dioxane library [273]. In A549 cells, tubacin caused a threefold increase of α-tubulin acetylation levels with no effects in cell morphology. In mouse embryonic stem cells, tubacin did not cause alterations in gene expression nor in cell cycle progression, supporting its specificity for the tubulin deacetylase HDAC6. Nevertheless, tubacin remains a preclinical tool compound due to its high lipophilicity, poor aqueous solubility and short *in-vivo* half-life/bioavailability [274].

Following the discovery of tubacin, another hydroxamic acid-based inhibitor was developed using structural homology-based optimization for higher HDAC6 selectivity, called Tubastatin A [275]. In primary cortical neuron cultures, Tubastatin A increased α-tubulin acetylation without marked elevation of histone acetylation at low concentrations. However, at higher doses, a slight induction of histone hyperacetylation was observed, consistent with increasing off-target HDAC activity. Tubastatin A has been widely used in cell and animal studies to dissect HDAC6-dependent phenotypes [276–278], but it remains a preclinical research inhibitor and has not advanced into clinical trials.

Further efforts to improve the pharmacokinetics and selectivity profiles of HDAC6 inhibitors led to the development of ricolinostat (ACY-1215) and citarinostat (ACY-241). Ricolinostat was first introduced in combination with bortezomib, a proteasome inhibitor, in the context of multiple myeloma treatment [279]. This study established ricolinostat as a potent and selective inhibitor of HDAC6 activity, increasing acetylated α-tubulin even at low doses without affecting histone acetylation. In mouse xenograft models injected with human multiple myeloma cells, treatment with ricolinostat suppressed solid tumor growth and increased acetylation of α-tubulin. Currently, ricolinostat has been tested in multiple phase I/II trials, both as monotherapy or in combination with other agents [280–283]. Citarinostat was later developed as a second-generation, more potent and selective follow-up to ricolinostat. Preclinically, the combination of this inhibitor or its predecessor with paclitaxel increased hyperacetylation of tubulin together with a higher frequency of multipolar spindles and subsequent cell death, further supporting the mechanistic rationale for testing ricolinostat/citarinostat plus taxanes in the clinic. Citarinostat is currently being explored in phase I clinical trials either alone or in combination for treating several cancers [251, 284–286].

Due to the undesirable features observed in hydroxamic acid-based HDAC6 inhibitors, such as poor pharmacokinetics, low selectivity profiles, and generation of active metabolites [274], Takeda researchers optimized high-throughput screening non-hydroxamic acid-derived hit compounds into two potent, selective HDAC6 inhibitors: T-3796106 and T-3793168 [287]. They both showed high selectivity for HDAC6 when compared to other HDAC isoforms. In murine superior cervical ganglion explants these compounds increased the levels of α-tubulin acetylation in a dose-dependent manner without causing cytotoxicity or morphological aberrations. To date, they remain preclinical tool compounds that require further pharmacokinetic optimization and *in vitro* characterization for clinical advancement.

In parallel, SIRT2-targeted inhibitors have also been developed to dissect tubulin-deacetylation pathways. In this context, SirReal inhibitors, discovered via high-throughput screening of an aminothiazole library, are the most relevant selective SIRT2 inhibitors to date [288]. By X-ray crystallography it was confirmed that SirReal2 binds specifically to the active site of SIRT2. Additionally, in HeLa cells, SirReal2 selectively hyperacetylated α-tubulin without increasing the acetylation of mitochondrial proteins, p53 or cell cycle distribution. Even though they remain preclinical research tools and have not entered clinical testing, these results validate their use as tools to dissect SIRT2 roles in cancer.

More recently, a first class of dual SIRT2/HDAC6 inhibitors has been developed as molecular tools to probe the functional consequences of tubulin hyperacetylation [289]. These hydroxamate-based hybrids were designed by linking SirReal-derived SIRT2 inhibitors with *N*-hydroxybenzamide HDAC6 inhibitors using a triazole-based linker. From this study, one potent candidate, Mz-325, exhibited high on-target selectivity, induced hyperacetylation in PC-3 M-prostate cancer cells, and reduced cell viability more effectively than the corresponding single inhibitors or simple combinations across a panel of cancer cell lines (HGC27, MCF-7, PC-3 M-Luc, and W1). Thus far, these dual SIRT2/HDAC6 inhibitors have not entered clinical testing and remain at the preclinical research stage, serving primarily as chemical probes for the role of tubulin-code deacetylation in cancer.

Beyond deacetylases, the tubulin code has been targeted from the “writer” side by putative inhibitors of αTAT1. Three 2,4-disubstituted thiazole derivatives identified in a small-molecule screening were reported to reduce tubulin-Lys40 acetylation in MDA-MB-321 cells without affecting α-tubulin expression and histone H3 acetylation [290]. Yet detailed structural and mechanistic evidence suggests that these compounds may primarily bind to the Lys40 region of tubulin rather than to αTAT1 itself. Although these molecules show antiproliferative effects in xenograft models, they remain experimental tools without any established clinical development status.

### Inhibitors of the tubulin tyrosination/detyrosination cycle

Pioneering studies have unveiled that parthenolide, a natural compound with anticancer activity, inhibits tubulin carboxypeptidases, reducing α-tubulin detyrosination levels [291]. Studies in HeLa cells confirmed that a high parthenolide concentration can partially inhibit tubulin detyrosination by binding to the active-site cysteine of VASH1 [292]. However, recent *in vitro* and *in cellulo* work has shown that parthenolide prevents the polymerization of microtubules by forming adducts on both cysteine and histidine residues on tubulin itself causing protein aggregation [293–295]. Parthenolide was also recently shown to bind to the kinetochore protein ZNF207/BUGZ, thereby impairing the establishment of proper kinetochore-microtubule attachments [296]. These new findings suggest an indirect effect of parthenolide on α-tubulin detyrosination. Due to these findings and the fact that parthenolide interacts with many intracellular targets such as NF-kB, DNA methyltransferase and focal adhesion kinase [293, 294], it should be regarded as a non-selective compound.

More recently, potent VASH inhibitors have been developed, such as EpoY and LV80. By mimicking the C-terminal tyrosine of α-tubulin, EpoY covalently targets the catalytic cysteine 169 in VASH1/2 through its epoxide group, thereby blocking detyrosination [297]. Preclinical studies have shown that EpoY shows improved selectivity towards VASH-family detyrosinases compared with earlier compounds and has been widely used to study detyrosination-dependent phenotypes, including megakaryocyte differentiation and maturation [298], EMT in lung cancer cells [205], and neural differentiation pathways [52]. LV80, which shows superior efficiency in inhibiting VASH activity both *in vitro* and *in cellulo,* along with favorable effects *in vivo* [205], has enabled a deeper investigation into the role of α-tubulin detyrosination in maintaining mesenchymal traits in cancer by increasing E-cadherin levels [205]. Concurrently, optimized analogs such as LV43, LV80 and LV87 were synthesized to improve affinity, bioavailability, chemical and biological stability, cell penetration and potency [299]. Although these analogs are active in the nanomolar range and appear very promising, no clinical studies have yet been reported.

On the opposing side of the tyrosination/detyrosination cycle, only one study has described sesterterpene lactones from *Salvia dominica* as putative TTL inhibitors [300]. Compound-immobilized affinity chromatography and surface plasmon resonance showed that 18 of 24 isolated compounds bound and interacted with TTL directly. Notably, the most active compound penetrated MCF-7 cells and increased Δ2-tubulin levels, suggesting TTL inhibition and a consequent blockage of tubulin re/tyrosination. Besides these results, no subsequent mechanisms or translational studies have been reported on this compound.

### Inhibitors of the tubulin polyglutamylation/deglutamylation cycle

Pharmacological modulation of tubulin polyglutamylation remains relatively unexplored compared to other tubulin PTMs. To date, the earliest and best-characterized inhibitors are phosphinic acid-based pseudo-dipeptides [301] designed to simultaneously target glutamylating and deglutamylating enzymes. These compounds decreased mouse TTLL7 activity *in vitro* from a millimolar to a micromolar range. However, the original study did not include *in cellulo* validation, and there is still no clear published cellular follow-up characterization of these compounds as effective intracellular deglutamylation inhibitors.

In another recent study, LDC10 was also identified as a putative TTLL inhibitor through a high-throughput screening campaign [302]. HEK293T cells treated with LDC10 showed significant reduction of both mono- and polyglutamylation, and LDC10 blocked the glutamylating activity of multiple TTLL family members, from initiases TTLL4 and 7 to elongases TTLL11 and 6. Currently, this compound is under medicinal chemistry-based optimization to enhance specificity and improve lead- and drug-likeness.

In a parallel endeavor, 2-phosphonomethylpentanedioic acid (2-PMPA) was found to efficiently inhibit CCP activities required for tubulin deglutamylation [303]. More specifically, 2-PMPA inhibited recombinant CCP1 deglutamylation activity on tubulin *in vitro* with an IC_50_ of 0.21 mM. This work thus identified the first inhibitor of tubulin deglutamylases, but its efficiency *in cellulo* remains to be demonstrated.

In contrast to the HDAC6-targeted compounds, which have entered clinical testing, inhibitors of the VASH1/2-SVBP complex, TTL, and TTLL-mediated polyglutamylation remain at the discovery or preclinical stage and have not yet been evaluated in human trials. These pharmacological agents and their current development stage are summarized in Table 2, which distinguishes clinically approved or trial-enrolled compounds (primarily HDAC-directed) from preclinical tools targeting acetylation/deacetylation, tyrosination/detyrosination, and polyglutamylation/deglutamylation.

**Table 2:** Summary of available pharmacological inhibitors of tubulin code “writers” and “readers” relevant for paclitaxel sensitivity

Enzyme class	Target	Compound name	Selectivity Profile	Clinical/Development Status	Refs
Deacetylase	Class I/II HDACs	Vorinostat (SAHA)	Pan‑HDAC inhibitor	FDA-approved; multi-trial clinical use	[265–267] [271]
Deacetylase	Class I/II HDACs	Trichostatin A (TSA)	Pan- HDAC inhibitor	Preclinical tool; narrow therapeutic index	[315, 316]
Deacetylase	Class I/II HDACs	Valproic acid (VPA)	Pan- HDAC inhibitor	Approved drug; repurposed for oncology	[269, 270]
Deacetylase	HDAC6	Tubacin	First‑generation selective HDAC6 inhibitor	Preclinical research tool	[273, 274]
Deacetylase	HDAC6	Tubastatin A	Optimized hydroxamate; selective HDAC6 inhibitor	Preclinical research tool	[275–278]
Deacetylase	HDAC6	Ricolinostat (ACY‑1215)	Selective HDAC6 inhibitor	Phase I/II and Phase II clinical trials	[279–283]
Deacetylase	HDAC6	Citarinostat (ACY‑241)	Selective HDAC6 inhibitor	Phase Ib/early Phase II clinical trials	[251, 284, 286]
Deacetylase	HDAC6	T‑3,796,106	Selective non‑hydroxamic HDAC6 inhibitor	Preclinical research tool	[287]
Deacetylase	HDAC6	T‑3,793,168	Selective non‑hydroxamic HDAC6 inhibitor	Preclinical research tool	[287]
Deacetylase	SIRT2	SirReal2	Selective SIRT2 inhibitor	Preclinical research tool	[288]
Deacetylase	SIRT2/HDAC6	Mz‑325	First‑in‑class dual Sirt2/HDAC6 inhibitor	Preclinical research tool	[289]
Deacetylase‑related	αTAT1/MT lattice	2,4‑ disubstituted thiazoles	Non-selective; tubulin‑lattice binding	Preclinical/experimental	[290]
Carboxypeptidase	VASH1/2‑SVBP	Parthenolide	Non‑selective; covalent tubulin ligand	Preclinical tool	[291–296, 317]
Carboxypeptidase	VASH1/2‑SVBP	EpoY	Selective VASH inhibitor	Preclinical	[52, 205, 295, 297, 298]
Carboxypeptidase	VASH1/2‑SVBP	LV43, LV80, LV87	Optimized EpoY‑type analogs	Preclinical lead-optimization	[205, 299]
Ligase	TTL	*Salvia dominica* sesterterpenes	Experimental TTL inhibitors	Preclinical/experimental	[300]
Glutamylase	TTLL family	Phosphinic acid‑ based pseudo‑ dipeptides	TTLL7- focused inhibitor	Preclinical biochemical tool	[301]
Glutamylase	TTLL family	LDC10	Novel Pan-TTLL inhibitor	Preclinical lead‑optimization	[302]
Deglutamylase	CCP1	2-PMPA	First-in-class CCP inhibitor	Preclinical research tool	[303]

## Conclusions, challenges and outlook

**Fig. 4:**
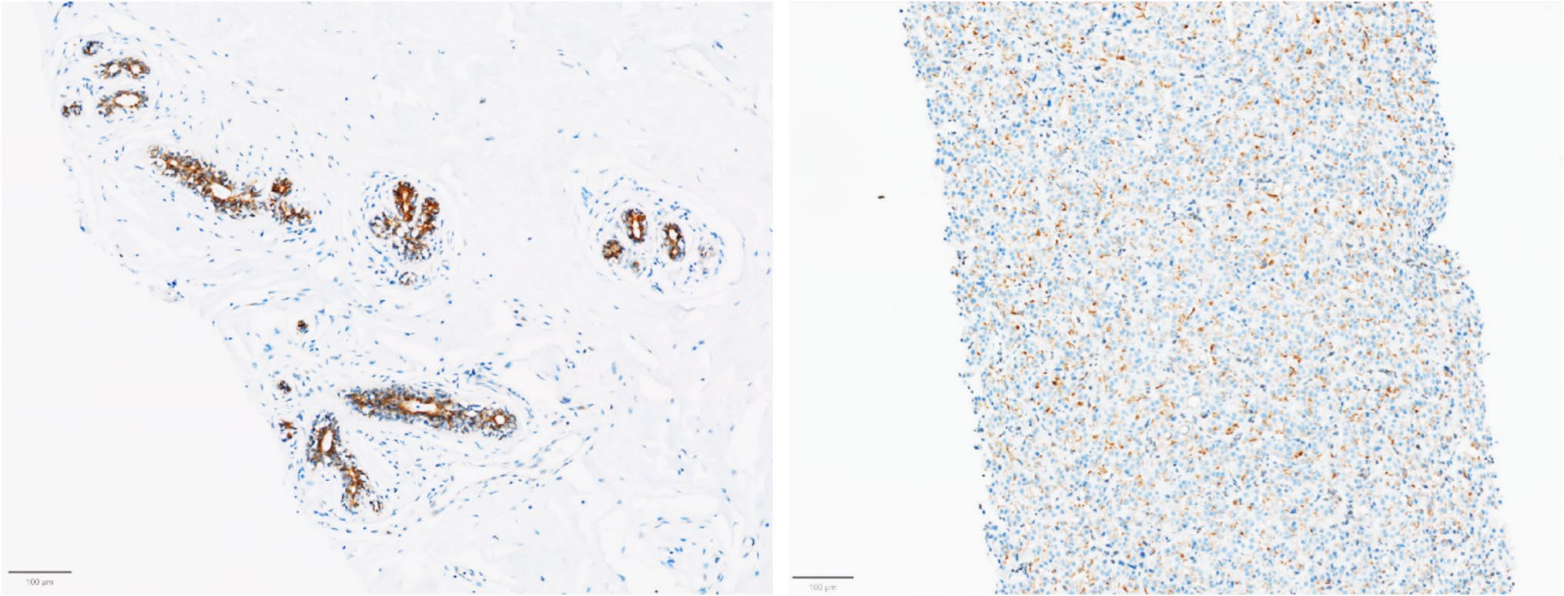
IHC detection of α-tubulin acetylation (AcK40) in FFPE biopsy tissue samples. Representative image showing positive DAB staining, detecting acetylated α-tubulin in normal (left) and tumoral (right) human breast tissue samples. DNA counterstained in blue using hematoxylin

This review highlights a new direction for precision oncology, emphasizing the importance of developing specific therapeutic strategies for cancer patients with poor prognosis, especially those that develop resistance to MTAs, such as taxanes. Currently, there are no clinically validated biomarkers that can effectively predict taxane response/resistance in cancer patients. Proper antibody validation is usually a rate-limiting factor for translating a biomarker from research into clinical practice. Even when findings are reproducible, turning a biomarker into a routine clinical tool needs a well-thought strategy: it should provide clear added value in patient stratification, and easily integrate into established testing routines, for instance through immunohistochemistry (IHC) on formalin-fixed paraffin-embedded (FFPE) tissue biopsy samples commonly used for histopathological confirmation [304, 305]. Currently, efforts to validate tubulin PTMs, such as α-tubulin acetylation, as potential predictive biomarkers for taxane response in breast cancer are incorporating such guidelines to develop valuable tools for breast cancer patient stratification and avoid unnecessary side effects (Fig. 4) [306].

Furthermore, the findings highlighted here underscore the need for a deeper understanding of the molecular mechanisms underlying paclitaxel cytotoxicity, particularly its relationship with α-tubulin acetylation, detyrosination and polyglutamylation, potentially unveiling new therapeutic vulnerabilities and supporting the development of novel agents, including VASH, TTL and TTLL inhibitors that can be further applied as therapeutic adjuvants of MTAs to reduce/overcome chemoresistance. Targeting the cancer tubulin code may represent an alternative or complementary approach to conventional therapies with MTAs, with the potential to fine-tune microtubule behavior and improve treatment outcomes in selected cancer contexts.

## Data Availability

No datasets were generated or analyzed during the current study.

## Abbreviations

αTAT1: α-Tubulin acetyltransferase 1
EMT: Epithelial-to-mesenchymal transition
HDAC: Histone/tubulin deacetylase
SIRT: Sirtuin
TTL: Tubulin-tyrosine ligase
VASH: Vasohibin
CENP-E: Centromere-associated protein E
MCAK: Mitotic centromere-associated kinesin
TTLL: Tubulin-tyrosine ligase like
CCP: Cytosolic carboxypeptidases
